# Quantification of tremor severity with a mobile tremor pen

**DOI:** 10.1016/j.heliyon.2020.e04702

**Published:** 2020-08-19

**Authors:** Tibor Zajki-Zechmeister, Mariella Kögl, Kerstin Kalsberger, Sebastian Franthal, Nina Homayoon, Petra Katschnig-Winter, Karoline Wenzel, László Zajki-Zechmeister, Petra Schwingenschuh

**Affiliations:** a Department of Neurology, Medical University of Graz, Auenbruggerplatz 22, Graz, 8036, Austria; b Tremitas GmbH, Schleppe-Platz 5/1/2, 9020, Klagenfurt, Austria

**Keywords:** Accelerometer, Essential tremor, Mobile monitoring, Parkinson's disease, Pen-shaped sensor, Tremor, Biomedical engineering, Biomedical devices, Neurology, Clinical research, Diagnostics, Biomarkers

## Abstract

**Background:**

An objective evaluation of tremor severity is necessary to document the course of disease, the efficacy of treatment, or interventions in clinical trials. Most available objective quantification devices are complex, immobile, or not validated.

**New method:**

We used the TREMITAS-System that comprises a pen-shaped sensor for tremor quantification. The Power of Main Peak and the Total Power were used as surrogate markers for tremor amplitude. Tremor severity was assessed by the TREMITAS-System and relevant subscores of the MDS-UPDRS and TETRAS rating scales in 14 patients with Parkinson's disease (PD) and 16 patients with Essential tremor (ET) off and on therapy. We compared tremor amplitudes assessed during wearable and hand-held constellations.

**Results:**

We found significant correlations between tremor amplitudes captured by TREM and tremor severity assessed by the MDS-UPDRS in PD (r = 0.638–0.779) and the TETRAS in ET (r = 0.597–0. 704) off and on therapy. The TREMITAS-System captured the L-Dopa-induced improvement of tremor in PD patients (p = 0.027). Tremor amplitudes did not differ between the handheld and wearable constellation (p > 0.05).

**Comparison with existing methods:**

We confirm the results of previous studies using inertial based sensors that tremor severity and drug-induced changes of tremor severity can be quantified using inertial based sensors. The assessment of tremor amplitudes was not influenced by using a handheld or wearable constellation.

**Conclusions:**

The TREMITAS-System can be used to quantify rest tremor in PD and postural tremor in ET and is capable of detecting clinically relevant changes in tremor in clinical and research settings.

## 1 Introduction

Tremor is defined as an involuntary, rhythmic, oscillatory movement of a body part and is classified along two main axes – clinical features and etiology [1]. It is considered as the most common movement disorder [1] and a population-based study in elderly people above the age of 50 years showed the highest prevalence for increased Physiological Tremor (9.5%), followed by Essential Tremor (ET) (3%) and Tremor in Parkinson's Disease (PD) (2%) [2]. Tremor in PD is characterized by a 4–6Hz, regular, asymmetrical resting tremor (most often affecting the hands), accompanied by bradykinesia (±rigidity), while ET is characterized by a bilateral action tremor of the upper limbs which may be accompanied by tremor of the head, voice, or lower limbs [1].

The evaluation of tremor severity is essential in order to assess the patients’ disease status and progression and the effect of treatment. Information captured by qualitative means may be useful for pragmatic management and planning, but quantification is necessary for precise monitoring and research. Two kinds of measures can be used for tremor evaluation. The first is subjective, inferential, based on rater-based interview and examination or patient self-assessment, and consists of rating scales and questionnaires. The second type of measure is objective, factual, based on technology-based devices equipped with one or more types of transducers converting a physical property of tremor into an electrical signal [3, 4]. Various transducer-based methodologies such as accelerometry, electromyography, gyroscopy, electromagnetic tracking, actigraphy, and digitizing tablets are currently used [3]. Due to their size, their weight, potential high costs, time consuming measurements and their complexity, most devices are only used in electrophysiological laboratories and are not as yet applicable for daily clinical use or home monitoring. Furthermore, the diagnostic accuracy has not been evaluated in large prospective clinical trials for most technology-based devices and different setups and criteria have not been standardized [4].

For this study, we used the Tremitas-System (TREM/Tremitas GmbH, Klagenfurt, Austria), an easy to use combined hardware and software tremor quantification system. The aims of the trial were to demonstrate the usefulness of TREM relating to the following aspects: (1) Is TREM capable of objectively quantifying rest tremor in PD and postural tremor in ET. (2) Can TREM be used to detect changes in tremor severity comparing the off and on drug status of patients with PD and ET in clinical practice. (3) Do tremor amplitudes differ if TREM is used in a handheld or wearable constellation. Furthermore, existing challenges in sensor technologies and signal processing were assessed and solutions suggested.

## 2 Methods

### 2.1 Patients

We consecutively recruited 14 patients with tremor in PD (mean age: 68.6; range, 55–78; 5 women) and 16 patients with ET (mean age: 65.4; range, 29–79; 5 women) from the movement disorder outpatient clinic at the Medical University of Graz, Department of Neurology. The inclusion criteria for PD patients were a diagnosis of PD following the Queen Square Brain Bank diagnostic criteria [5], presence of a rest tremor in the morning before intake of PD related medication and absence of levodopa-induced dyskinesia according to patients’ history and medical records. ET patients were diagnosed according to the Consensus statement of the Movement Disorder Society on tremor for ET [1].

The average clinical visit lasted for 2 h. PD patients were required to stop the intake of all tremor influencing medications 12 h before a clinical examination (Practical OFF-state). Demographic data and information about the onset of the disease, the disease duration, previous and current medications were gathered from all patients. After a proper clarification, each patient signed the informed consent to join the clinical trial. The trial was approved by the local ethics committee of Graz and the Austrian Federal Office for Safety in Health Care (AGES, BASG).

In PD patients, all clinical and instrumental tests were made before medication intake (Baseline/OFF state) and one hour after medication intake in an ON state. PD patients received 1.5 times the L-dopa equivalence dose of their standard morning dose of the individual anti PD medication, which was Levodopa/Benserazid- MADOPAR „Roche“ 100/25mg-lösliche Tabletten® (soluble tablets) corresponding to a L-Dopa challenge test [6]. ET patients were assessed 60 min after intake of their standard morning dose of individual anti tremor medications, which was propranolol from 10 to 40mg and/or primidone from 120 to 250mg (ON state).

The patients' disease severity was evaluated with the Movement Disorder Society (MDS)-sponsored revision of the Unified Parkinson's Disease Rating Scale (MDS-UPDRS) for PD [7] and the Essential Tremor Rating Assessment Scale (TETRAS) [8]. Demographic and clinical details are summarized in Table 1.

**Table 1:** Demographic Details and Clinical Scores assessed at baseline.

Characteristics	Parkinson's Disease	Essential Tremor
Sex (m/f)	9/5	11/5
Age: Mean (SD)	68.6 (7.86)	65.4 (11.69)
Age of disease onset: Mean (SD)	63.2 (7.21)	41.7 (24.91)
Disease Duration: Mean (SD)	5.4 (2.6)	23.7 (20.00)
MDS UPDRS III: Mean (SD)	35.6 (8.45)	NA
TETRAS Performance: Mean (SD)	NA	18.8 (5.25)
MDS-UPDRS 3.17 + 3.18; more affected hand: Mean (SD) [Range]	4.1 (1.2) [2–6]	NA
TETRAS 4a R + L: Mean (SD) [Range]	NA	2.6 (1.2) [1–4.5]
Daily Levodopa Equivalence Dose: Mean (SD) [Range]	471.64 (291.78) [52–1246]	NA

Description: SD = Standard deviation; MDS UPDRS III = Movement Disorders Society Unified Parkinson's Disease Rating Scale, Part III Motor function at Baseline; TETRAS Performance = The Essential Tremor Rating Scale Performance Part at Baseline; Baseline = 12 h after the last intake of anti-tremor medication; NA = not applicable.

### 2.2 Hardware and sensors

**Figure 1:**
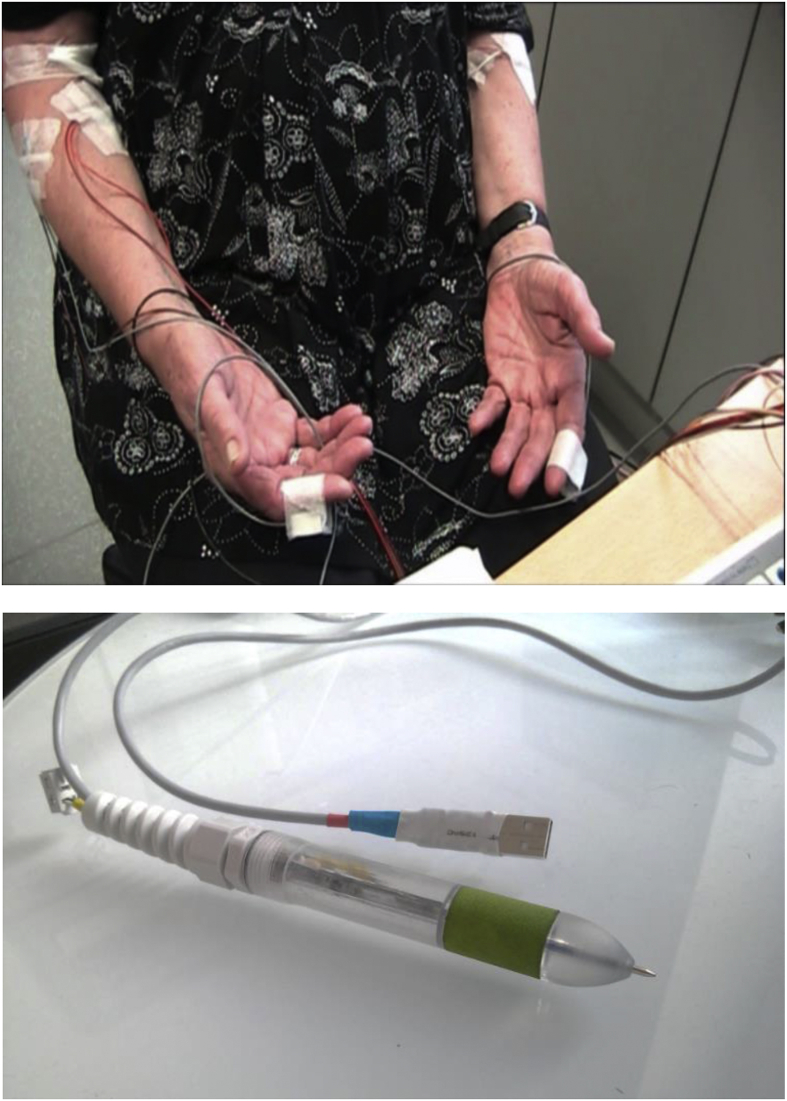
EMG-Accelerometer (top) and TREM (bottom) in comparison; the pen-shaped sensor can be held by patients and needs only one cable.

TREM consists of a two-sided electronic printed circuit board (PCB), a two-part plastic case and a connection cable. The form of the case resembles a pen; it has a length of 180 mm, the average diameter of the pen is 18 mm. The pen-shaped form was chosen to increase the usability; by telling patients to hold TREM like a typical pen, wrong grip positions can be reduced, and usability errors minimized. An unscrewable chamber at the front end of the case makes it possible to insert a shortened ball pen refill at the tip of the case. At the grip zone of the pen, a detachable plastic element is mounted for a better grip. Additionally, this plastic element is an indicator where to hold TREM. At the back end of the case, the PCB can be inserted exactly and jitter-free inside the case; an unscrewable strain relief closes the back end of the case. The PCB consists of a 9-dimensional inertial system (3D Accelerometer, 3D Gyroscope, 3D Magnetometer/Invensense MPU9250), a microcontroller (TI MSP430F5172IRSB) and auxiliary components for the power supply and the data transmission. The inertial system is mounted close to the grip zone, so that the distance between the tremor producing fingers and the sensors is as short as possible to reduce the risks of artifacts. Accelerometers and gyroscopes are accepted sensors for tremor quantification [3]. The magnetometer is a supporting sensor, which reacts to changes of the earth's magnetic fields. It is not suitable for tremor quantification, but combined with the other sensors, it can provide information about the three-dimensional position of TREM during writing and drawing activities. The elastic connection cable has a diameter of 4mm and a length of approximately 1.5 m. This cable serves as a power supply and as an interface for serial data transmission. TREM has a total weight of approximately 50 g, without the connection cable, it has a weight of approximately 30 g. The weight is chosen to be as low as possible so that holding TREM does not cause an active muscle contraction. If a higher weight is used, the pathological rest tremor could disappear, and the tremor signal could be distorted. The hardware was designed so that preparation and measurement activities can be efficiently done; the local EMG-accelerometer was chosen to be the benchmark for comparison (see Figure 1).

The TREM sampling rate was set to 100Hz, the detectable acceleration was set to +/- 4g. The gyroscope is set to detectable rotations of +/- 500°/s. No special filters were activated. The TREM sensors send continuous data in real-time to a PC via the serial interface. TREM is connected to the PC via a USB interface.

**Figure 2:**
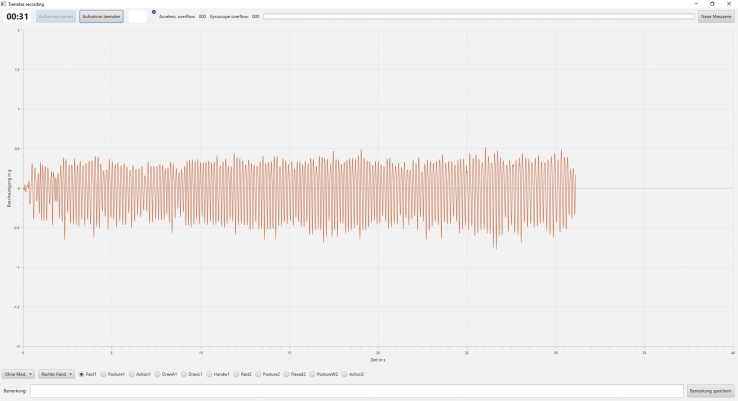
Tremitas Recording Software during a measurement; the main area shows the tremor amplitude in g and the course of time in seconds; Users can start and stop measurements manually, insert comments to current measurements, name measurements and store a data set after an implemented measurement.

The data generated by TREM are received and stored by the Tremitas Recording Software (Tremitas GmbH/Version 1.0) (see Figure 2).

### 2.3 Recording protocol and documentation

The measurement process was divided into two phases. During phase 1, patients were off anti-tremor medication for at least 12 h (OFF/Baseline state). Patients were seated on a chair and depending on the measurement position, TREM was actively held by the patients like a regular pen (“handheld condition”; Montage M1) or it was attached to their index finger and their back of the hand with adhesive tapes (“wearable condition”; Montage M2). All patients with PD and ET underwent the same protocol (see Table 2). However, in PD we were mainly interested in rest tremor and in ET patients in postural tremor.

**Table 2:** Measurement protocol.

Activity	Montage	Duration
Rest Tremor	M1 and M2	30s and 90s
Postural Tremor	M1 and M2	30s and 90s
Action Tremor	M1 and M2	30s and 90s
Spiral Drawing	M1	30s
Line Drawing	M1	30s
Sentence Writing	M1	30s
Wing-beating Tremor	M2	90s
Postural Tremor with Weight	M2	30s

Description: Rest Tremor = Hands are positioned on the thighs, relaxed sitting position; Postural Tremor = Both arms are extended forward and the fingers are spread, the arms are perpendicular to the torso; Action Tremor = Fingertip/Tip of the pen is moved between the own nose and another person's index finger; Spiral Drawing = The ball pen refill is used to draw and trace a preprinted Archimedean spiral on a piece of paper; Line Drawing = a straight line is drawn between two preprinted frames; Sentence Writing = An example sentence is written and repeated for 30 s; Wing-beating Tremor = Hands are positioned in front of the chest and the fingers are showing towards each other and almost touch each other – the elbows are angled and positioned at shoulder height; Postural Tremor with Weight = A 0.5kg weight is mounted on the patient's wrist and the postural tremor position is implemented; Montage 1 (M1) = Patient is holding TREM like a pen on their own or are writing with TREM; Montage 2 (M2) = TREM is attached to the patient's index finger and their back of the hand.

The protocol was implemented in random order and performed for the right and the left hand. In PD patients, approximately 60 min after medication intake (ON State), the second measurement phase was initiated following the same measurement protocol.

The objective and clinical assessment were recorded and documented simultaneously during each measurement position. TREM sent continuous accelerometer, gyroscope and magnetometer values to the PC. A clinical expert was rating the patients according to the MDS-UPDRS and the TETRAS [8], [7][7, 8].

### 2.4 Data pre-processing

The received data, which was sent via the serial interface, were preprocessed, so that a standardized comparison of data sets is possible.

The software gathers the raw data and converts the binary acceleration, rotation and orientation information into g, °/s and μT values. It is possible to manually start and stop a measurement with the software, the user can decide how long a measurement should take; nevertheless, the software was programmed to take measurements of 30, 60 or 90 s, depending on the measurement position, but allowed longer measurement periods. A visual indicator shows if the recommended time length for a measurement position is reached. An internal control mechanism checks whether single measurement data points were lost or not. For each measurement, the software creates a CSV file with the raw data. These values are then used to implement a spectral analysis and to extract the relevant tremor parameters. The software does not apply special filtering or optimizing algorithms, only a general Discrete Fourier Transformation (DFT) is used for spectral analysis.

### 2.5 Calculated values and statistical analysis

For each measurement position, a TREM data set was created. A data set consists of 3,000 to 9,000 single TREM measurement points, depending on the length of a measurement. To minimize selection bias we decided to use the first 30 s for each data set. The data sets were transformed via DFT using MATLAB software. The DFT's buffer size was set to 200 samples, making a spectral resolution of 0.5Hz possible.

A cut-off was implemented for the resulting frequency spectrum and all frequency elements below 3 Hz and above 20 Hz were ignored (Cut-Off procedure). The lower frequency elements, which are cut-off, contain the earth's gravity (1g) and slow active movements, which can influence tremor measurements [3]. The upper frequency elements, which are cut-off, contain no human tremor anymore, only noise [9]. This step was implemented for each of the three accelerometer and gyroscope dimensions.

The remaining frequency spectra were used to extract 3 clinically accepted tremor parameters [10]: The tremor amplitude and the signal energy as a parameter for tremor severity and the tremor frequency as a parameter for tremor velocity. The tremor amplitude (Power of Main Peak, PMP) is defined as the amplitude value (g-value) of the main peak within a given frequency spectrum [11]. The tremor frequency (Peak Frequency, PF) is the first dominant peak's frequency in the frequency spectrum. The signal energy of the tremor (Total Power, TP) is the integrated signal energy of the frequency spectrum between the cut-off frequencies (For TP calculation, cut-off frequencies of 1 and 30Hz were used). The parameters were calculated for each of the accelerometer's dimensions (X, Y, Z); afterwards, the mean value ((X + Y + Z)/3) was calculated to implement a reduction from three to one dimension. These one-dimensional values were then used for further analysis.

For each measurement position and OFF/ON constellation, the three tremor parameters were calculated and further stored in a CSV/Excel file. These parameters where supplemented with demographic data and data from the clinical scales (TETRAS, UPDRS). Altogether, 416 data sets were analyzed. Data analysis was implemented via Tremitas Software and MATLAB, further analysis was made with SPSS 17.0.

Pearson Correlations were used to compare the objective measurement results of TREM with the rating score assessments of the clinical gold standard. For each TREM measurement result, a tremor score of one of the standardized tremor scales was provided. The UPDRS was used for PD and TETRAS was used for ET. A comparison of the main parameters tremor amplitude and tremor frequency was implemented with clinical scales and between both groups (PD and ET) via t-tests. Changes of the main parameter tremor amplitude within the PD group before (OFF) and after (ON) medication intake were evaluated via paired t-tests. A p-value of 5% or lower equals a significant result.

Depending on the patients‘ diagnosis, different comparison criteria were defined for the correlation analysis. The PMP and TP results were compared to the following scales and subscores: For PD, the subscores of the UPDRS part III elements 3.17 (Tremor intensity) and 3.18 (Tremor continuity) were added. The relevant measurement positions were rest tremor, montages M1 and M2 were investigated and for each patient, the more affected side (MAS) was analyzed. The rest tremor measurements of the 14 PD patients with each montage were chosen as correlation groups. For ET, the score of TETRAS section 4a (Tremor intensity) was chosen. The relevant measurement positions were the postural tremor, montages M1 and M2 were investigated and the subscores for the left hand and the right hand were added. The postural tremor measurements of the 16 ET patients with each montage were chosen as correlation groups. Both montages were correlated for Baseline/OFF and ON states for the PD group and for ON states only for the ET group.

It is known from available literature that tremor amplitudes measured by accelerometers logarithmically correlate with clinical scales [12, 13]. This is due to the nature of the tremor intensity scores, which increase in a non-linear way with each point. Therefore, the PMP and TP measurement results are logarithmized (Base 10) and then compared to the corresponding scale scores. For the UPDRS, the PMP or TP results of the MAS were logarithmized (log(milli-g MAS)) and compared with MDS-UPDRS III 3.17 + 3.18. For TETRAS, the PMP or TP results for the left and the right side were logarithmized individually and then added (log(L) + log(R)) and compared with TETRAS-Performance point 4a R + L.

## 3 Results

No technical malfunctions or complications occurred during the clinical trial. Furthermore, no adverse effects were detected. At baseline all PD patients presented with a rest tremor (MDS-UPDRS 3.17 + 3.18; more affected hand: mean = 4.1, SD = 1.2, range = 2–6) and no patient developed levodopa induced dyskinesias in the ON state. All patients with ET presented with a postural tremor (TETRAS 4a R + L: mean = 2.6, SD = 1.2, range = 1–4.5) in ON state.

### 3.1 Comparison between TREM and clinical scales in both constellations

The measurements of montages 1 and 2 were analyzed via an independent t-test; the results are summarized in Table 3.

**Table 3:** Analysis Montage 1 vs. Montage 2.

Parameter	Significance p	Bonferroni corrected significance p
RT PMP MAS TREM OFF	0.707	1
RT PMP MAS TREM ON	0.947	1
RT TP MAS TREM OFF	0.253	1
RT TP MAS TREM ON	0.397	1
PT PMP ON	0.088	0.52
PT TP ON	0.086	0.516

Description: OFF = 12 h after the last intake of anti-tremor medication; ON = Status 60 min after the intake of; MAS = More affected side/the upper extremity (UE), which is more affected by tremor; PMP MAS OFF = Power of Main Peak of rest tremor of the more affected UE in Baseline state (logarithmized raw data); PMP MAS ON = Power of Main Peak of rest tremor of the more affected UE in ON state (logarithmized raw data); TP MAS OFF = Total Power of rest tremor of the more affected UE in Baseline state (logarithmized raw data); TP MAS ON = Total Power of rest tremor of the more affected UE in ON state (logarithmized raw data); RT = Rest tremor of PD patients; PT = Postural tremor of ET patients.

For the PD group, correlations between TREM (PMP and TP; OFF and ON) and UPDRS 3.17 + 3.18 are shown in Table 4. For the ET group, correlations between TREM (PMP and TP; ON) are shown in Table 5.

**Table 4:** Correlations TREM vs. UPDRS III 3.17 + 3.18

Parameter	Correlation r/significance p Montage 2	Correlation r/significance p Montage 1	Bonferroni corrected significance p Montage 2/Montage 1
PMP MAS TREM OFF	0.695/0.005	0.656/0.010	0.02/0.040
PMP MAS TREM ON	0.735/0.002	0.749/0.002	0.008/0.008
TP MAS TREM OFF	0.694/0.005	0.638/0.014	0.02/0.056
TP MAS TREM ON	0.703/0.005	0.779/0.001	0.02/0.004

Description: OFF = 12 h after the last intake of anti-tremor medication; ON = Status 60 min after the intake of 1.5 times the L-Dopa equivalence dose; MAS = More affected side/the upper extremity (UE), which is more affected by tremor; r = Pearson correlation coefficient; PMP MAS OFF = Power of Main Peak of rest tremor of the more affected UE in Baseline state (logarithmized raw data); PMP MAS ON = Power of Main Peak of rest tremor of the more affected UE in ON state (logarithmized raw data); TP MAS OFF = Total Power of rest tremor of the more affected UE in Baseline state (logarithmized raw data); TP MAS ON = Total Power of rest tremor of the more affected UE in ON state (logarithmized raw data).

**Table 5:** Correlations TREM vs. TETRAS-Performance point 4a R + L.

Parameter	Correlation r/significance p Montage 2	Correlation r/significance p Montage 1	Bonferroni corrected significance p Montage 2/Montage 1
PMP PT TREM ON	0.656/0.028	0.688/0.019	0.056/0.038
TP PT TREM ON	0.597/0.052	0.704/0.015	0.104/0.03

Description: ON = Status 60 min after the intake of the morning dose of anti tremor medication; PT = Postural tremor of the right and left upper extremity (sum); r = Pearson correlation coefficient; PMP PT ON = Power of Main Peak of postural tremor in ON state (logarithmized raw data); TP PT ON = Total Power of postural tremor in ON state (logarithmized raw data); TETRAS 4a R + L ON = TETRAS Performance Part –point 4 a right and left (Sum) in ON state.

The L-Dopa-challenge test [6] resulted in a significant tremor reduction in PD regarding clinical ratings and tremor amplitude (TP and PMP) recorded by TREM (See Table 6).

**Table 6:** Comparison of tremor severity before and after drug intake for PD.

	PMP RT MAS	TP RT MAS	UPDRS III 3.17 + 3.18
Significance	p = 0.027∗	p = 0.054	p < 0.001∗
Mean of data pair	1.09/0.72	0.002/-0.5	4.1/2.0
SD of data pair	0.6/0.5	0.97/0.88	1.2/1.9

Description: RT MAS = Rest tremor of the more affected upper extremity; UPDRS III 3.17 + 3.18 = MDS UPDRS Part III Subscores 3.17 + 3.18 (sum); PMP and TP values are provided in logarithmized form.

### 3.2 Further results with additional measurement positions and sensors

The main evaluation focus was set to the rest tremor for PD and the postural tremor for ET. Nevertheless, it was also possible to demonstrate good correlations for ET action tremor measurements (r > 0.600/TETRAS 4c). It was possible to extract relevant PFs from the drawing and writing positions, but the amplitudes were distorted due to the impact between TREM and the piece of paper on a desk while writing. The 9-dimensional inertial sensor was furthermore not able to reproduce the three-dimensional trajectory; writing, the written text, the drawn spirals and lines could not be digitally recreated with simple positioning algorithms.

The correlation analysis was additionally implemented with the measurements taken by the gyroscope. The average correlation over all measurement positions was 0.673 for the gyroscope and 0.699 for the accelerometer.

## 4 Discussion

Because of its high prevalence, clinicians of all specialties will encounter tremor in routine practice. The natural fluctuation of many pathological tremors makes judgement of any change of tremor severity challenging. Subjective measures for tremor evaluation consist of rating scales and questionnaires. In certain instances, an objective assessment of tremor via technology-based devices is needed.

In this study, we showed that TREM can quantify rest tremor in PD and postural tremor in ET and is capable of detecting changes in tremor amplitude induced by a L-Dopa challenge test [6] in PD.

The presented study and quantification approach to measure tremor in PD and ET go in-line with comparable and recently published studies. Smart watches, gloves, tablets, smart phones and body-worn multiple sensor solutions are used to detect and quantify tremor based on inertial sensors, especially accelerometers and gyroscopes [14, 15, 16, 17, 18, 19, 20, 21, 22, 23, 24, 25, 26, 27, 28, 29]. While other presented solutions focus on a wearable constellation, TREM was designed to be used as a handheld device. There was no significant difference when amplitudes of rest tremor in PD and postural tremor in ET patients were compared between a wearable and a handheld constellation. A handheld setup provides the following potential advantages: The device is not depending on individual anatomical features such as wrist or finger or belt sizes. Furthermore, it is not necessary to wear a handheld device for longer time periods; patient compliance for wearable systems could depend on the aesthetics and acceptance by the patient. To increase the compliance and usability rate, TREM was designed to be pen-shaped. The motivation for this was to develop a device, which resembles an everyday object so that patients are not confronted with an abstract object; this also included the commonly used wording “tremorpen” during the visits. When holding TREM, it was expected that patients intuitively grab the device correctly so that the risk of incorrect measurement positions is reduced, which was also the case. Finally, a handheld device is used during a task under a certain relevant condition, such as rest, posture, or action, which can be documented. Wearable systems often also track data if patients are pursuing activities of daily living and perform other tasks, which may not be relevant for tremor measurements. Although a handheld system, such as TREM, is not capable of providing a continuous monitoring solution, it can be an efficient device for tremor quantification at a given time under specified conditions.

Beside technical and clinical proof-of-concept approaches, general advantages and disadvantages of objective quantification tools in comparison to subjective tremor assessments need to be evaluated in future studies. Advantages of objective solutions are the availability of objective and quantifiable data, the possibility to receive long-term and continuous monitoring data and the availability of multiple technology platforms and sensors. However, disadvantages in comparison to subjective evaluations are potentially high costs of devices and solutions, often missing applicability in everyday clinical use, possible risks emerging from medical devices, missing large prospective clinical trials and missing standardization. Furthermore, objective devices are seen as supportive tools for clinical assessments, but not as replacements for daily examinations [3, 4].

An emerging field of interest is the home-monitoring of patients with PD to assess motor and non-motor symptoms to overcome the obstacles of single-point assessments and white coat effect [30, 31, 32, 33]. Additionally, long-term monitoring can provide summarizing data in between ambulatory assessments and more profound information about drug efficacy. While smart phones and similar devices are capable of such a monitoring constellation, the current technical setup of TREM is not suitable for such an approach. Further technological development is necessary, which will be subject to a next clinical study based on TREM technology.

A relevant technical consideration is the efficient peak frequency dimensional reduction. TREM captures human tremor in three dimensions and the DFT calculates three PFs (one for each dimension). For a clinical assessment, a single frequency value, which is derived from the three values, is easier to assess than 3 distinct values. However, it is not recommended to calculate this value by using the mean value ((PF_x_ + PF_y_ + PF_z_)/3) although this method is commonly used. The TREM measurement results showed frequently that tremor mainly manifests in two dimensions, while the third dimension is mostly noise with a random PF. If a reduced PF is calculated with two tremor-caused PFs and one random noise PF, this can distort the resulting average PF. As an example, a tremor measurement causes two PFs of 6Hz in two dimensions and a random noise PF is detected at 17Hz. The resulting incorrect PF would be 9.6Hz. Two alternatives are: (1) calculating the mean value of the two dimensions with the highest corresponding PMP and discarding the PF with the lowest PMP or (2) considering only the PF of the dimension with the highest corresponding PMP. TREM measurement results showed that if a significant peak and a PF were detected within the frequency spectrum of one dimension, then the same PF was detected in a second dimension. This finding makes it possible to consider just one PF.

Similar considerations are necessary for the efficient power of main peak dimensional reduction. Calculating the PF of a tremor measurement is generally less challenging than calculating and reducing three-dimensional PMP values to a one-dimensional value. Different methods are commonly used for tremor quantification, such as calculating a sum, calculating mean values and using norm values. There are two general approaches for this problem: A one dimensional PMP can be calculated by reducing three-dimensional amplitudes either before implementing a DFT or after. Both methods were applied on TREM measurements and the second method produced more stable results. The following considerations explain the drawbacks and risks of the first method: Calculating the reduced amplitude value before the DFT by using a mean value ((X + Y + Z)/3), or simply calculating the sum of the three values for each measurement point, is commonly used, but this method can distort the amplitude value by a factor of up to 100%. Using the mean value is a suitable way of calculation if the tremor manifests exactly along the sensor's axes, but in most real cases, tremor will not manifest along the axes.

**Figure 3:**
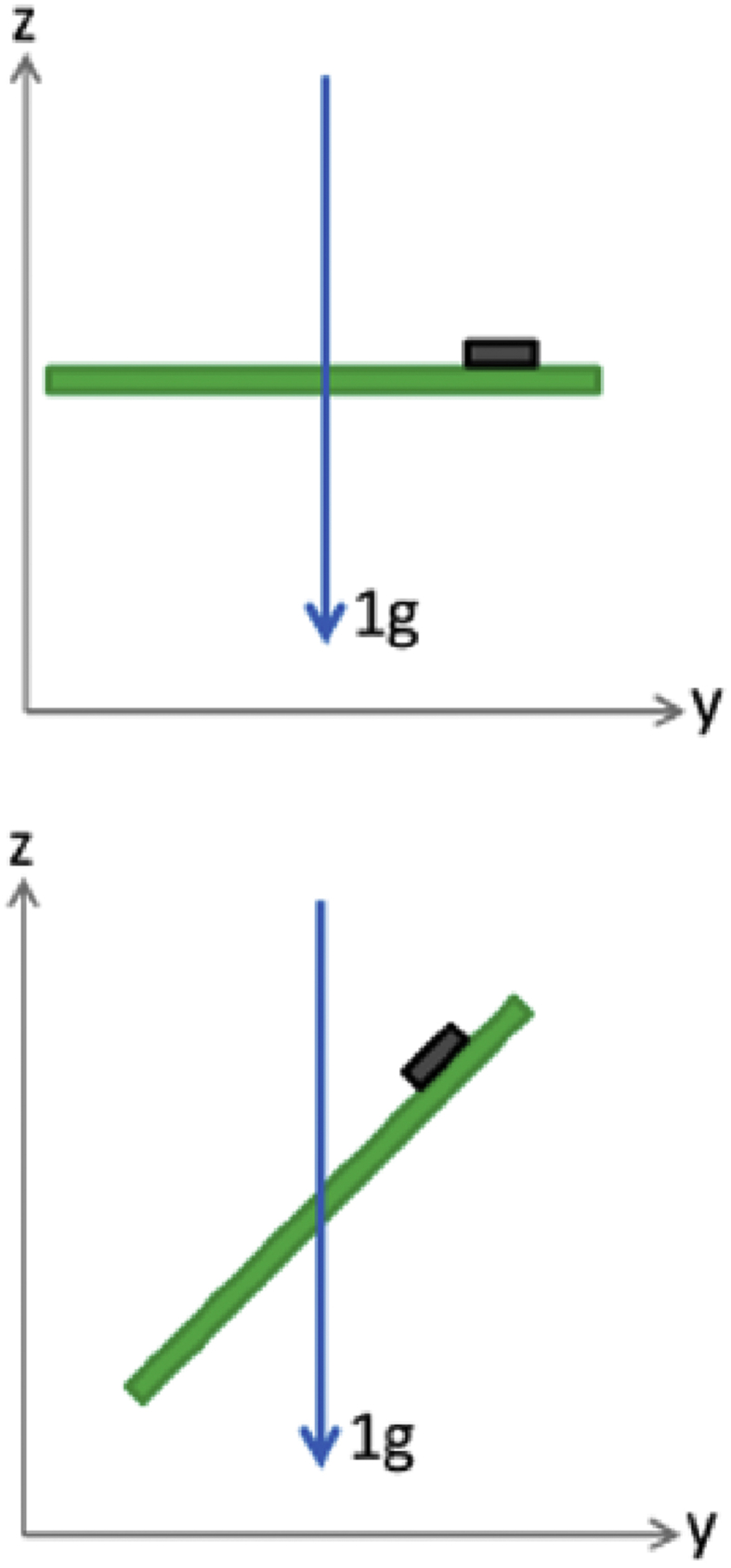
Examples of a 1g acceleration, which manifests along the sensor's axes (top) and in a 45° angle to the sensor's axes of a PCB (bottom).

In case one of Figure 3 (top), the sensor's axes would show the values [0 0–1]. Using the method of calculating the sum, the correct value is received (0g + 0g + (-1g) = -1g), by calculating the mean value, the result of 0.33g would be incorrect. In case one of Figure 3 (bottom), the sensor's axes would show the values [0 1/√2 -1/√2]. Using the method of calculating the sum, an incorrect value is received (0g + 1/√2g + (-1/√2g) = 0g), by calculating the mean value, the result would be again 0g. A suitable method of reducing this distortion is to calculate the norm of the spectral PMP values (√ (X^2^ + Y^2^ + Z^2^)). While this approach is recommended, we here used the averaged values for two reasons: With this first study we aimed to reproduce a widely used algorithm for processing accelerometer generated data. The measurement positions and especially the TREM angles could be supervised and corrected if needed. Therefore, a certain error compensation under supervision is possible, while this level of control would not be possible in a home environment. Additionally, while choosing a suitable accelerometer sensor for tremor quantification, the sensor's “Cross-Axis Sensitivity” should be considered beside noise levels and accuracies. A low cross-axis sensitivity lowers the distortions if a tremor does not manifest along the sensor's axes. The norm calculation is also recommended for the dimensional reduction after a DFT. If the transformed PMP values are added or the mean value is calculated, then a mathematical error factor of up to √3 can emerge.

While using the solutions described in the previous two paragraphs of this section can improve the quality of measured tremor data, additional considerations are necessary for further optimizations. Depending on the available hardware, a suitable buffer size needs to be chosen for the DFT. A buffer size of 200 seems to be appropriate for a sampling rate of 100 Hz, it provides a resolution of 0.5Hz, which should be sufficient for most scientific and clinical questions. A higher buffer size increases, a lower buffer size reduces the frequency resolution. TREM measurements did not provide clinically more relevant information when a resolution of 0.1Hz was applied. Choosing suitable DFT windowing functions is also a possibility to improve the measurement quality. Using a cut-off procedure is necessary to reduce the influence of the earth's gravity to a minimum. Tremor usually manifests in a frequency band between 3Hz and 15–20Hz, information below and above these thresholds only distort the results. An exception is possible if the harmonics of tremor peaks are to be analyzed. A tremor peak at 10Hz can have harmonic peaks at 20Hz and 30Hz, these peaks can in some cases be relevant for the TP.

TREM gathered tremor data with a 3D accelerometer and a 3D gyroscope. Selected PMP correlations were repeated with gyroscope amplitude data (rotation per second) after implementing a DFT. The results showed on average that the gyroscope PMP values provided better correlations in Montage 2 (TREM attached to hand) than the accelerometer PMP values, but worse correlations in Montage 1 (TREM held by patients). This indicates that a gyroscope may be a superior sensor for wearable quantification tools, but for handheld devices, the accelerometer is more suitable. Nevertheless, in several TREM measurements the gyroscope provided significant peaks when the accelerometer did not, and vice versa. Further investigations shall provide information how this complementary behavior can improve the tremor results.

PD rest tremor usually improves during muscle activation. A potential limitation of a handheld constellation was that rest tremor might be decreased in amplitude if TREM was actively held. TREM was developed to be as light-weight as possible so that it is possible to hold the system without eliminating rest tremor. Therefore, when recording rest tremor patients were instructed to hold TREM without any force and the back end of the pen shaped case was placed on the back of the hand. Although this was not tested as part of this clinical trial, observations showed that, as expected, tremor improved if patients had a stronger grip or started to move TREM. The weight of 30grams and the relaxed holding position make it possible to quantify rest tremor without eliminating the symptom.

Finally, Tremor PMP was found to be a suitable parameter to track levodopa induced changes of rest tremor in PD.

## 5 Conclusion

This clinical trial proves that TREM is capable of quantifying tremor in a clinical setting (assisted by trained personnel), while significantly correlating with relevant subscores of MDS-UPDRS and TETRAS for PD (rest tremor) and ET (postural tremor), respectively. These results confirm the validity of accelerometers, EMGs, Gyroscopes and digitizing tablets for tremor detection and tremor quantification [3]. The results further confirm that a therapeutic effect (improvement of tremor after drug intake) can be objectively captured via tremor amplitudes for PD. Regarding the small sample size, further research with a larger patient cohort is necessary to validate the presented results. Future clinical investigations will show if tremor can be captured in a home-environment and if it is possible to realize a home-monitoring possibility, similar to blood-pressure measurements.

## 6 Outlook

TREM will be further developed to increase its mobility by executing tremor calculations within the device and not on an external PC system. This would increase mobility and make it possible to use the device in clinical- and also home-environment settings. Future studies are planned to execute home-monitoring studies and to further identify suitable tremor parameters, which can be used to additionally distinguish different tremor types and also support differential diagnostic procedures. Current publications indicate that suitable algorithms are available for this aim [34].

## Declarations

### Author contribution statement

Tibor Zajki-Zechmeister: Performed the experiments; Analyzed and interpreted the data; Contributed reagents, materials, analysis tools or data; Wrote the paper.

Mariella Kögl, Kerstin Kalsberger, Sebastian Franthal, Nina Homayoon, Petra Katschnig-Winter, Karoline Wenzel: Conceived and designed the experiments; Performed the experiments; Wrote the paper.

László Zajki-Zechmeister: Analyzed and interpreted the data; Contributed reagents, materials, analysis tools or data; Wrote the paper.

Petra Schwingenschuh: Conceived and designed the experiments; Performed the experiments; Analyzed and interpreted the data; Wrote the paper.

### Funding statement

This work was supported by the 10.13039/501100004955Austrian Research Promotion Agency (Basic Program, FFG-Nr: 853136), the Carinthian Economic Assistance Fund (Complementary Grant Funding to the FFG Basic Program) and the Tremitas GmbH.

### Competing interest statement

Tibor Zajki-Zechmeister is Managing Director of Tremitas GmbH, working on a regular basis for the company and receives regular income. László Zajki-Zechmeister is a shareholder of Tremitas GmbH, not working on a regular basis for the company and does not receive income. The clinical trial was sponsored by the Tremitas GmbH; The sponsor provided the device and the auxiliary tools and provided the budget for performing the clinical trial to the Medical University of Graz where it was conducted. Mariella Kögl, Kerstin Kalsberger, Sebastian Franthal, Nina Homayoon, Petra Katschnig-Winter, Karoline Wenzel and Petra Schwingenschuh (principal investigator) work on a regular basis at the Medical University of Graz. This clinical study was designed as a research project, thus receiving funds from the Austrian Research Promotion Agency. Tibor Zajki-Zechmeister and László Zajki-Zechmeister contributed to the technical parts of the study design and technical data analysis only. As principal investigator Petra Schwingenschuh designed the clinical study and was responsible for data collection, data analysis, interpretations, and conclusions, while the members of Tremitas GmbH did not have any influence on these parts of the study.

### Additional information

No additional information is available for this paper.
